# Small RNAs induce the activation of the pro‐inflammatory TLR7 signaling pathway in aged rat kidney

**DOI:** 10.1111/acel.12629

**Published:** 2017-06-30

**Authors:** Eun Kyeong Lee, Ki Wung Chung, Ye Ra Kim, Sugyeong Ha, Sung Dae Kim, Dae Hyun Kim, Kyung Jin Jung, Bonggi Lee, Eunok Im, Byung Pal Yu, Hae Young Chung

**Affiliations:** ^1^ Molecular Inflammation Research Center for Aging Intervention (MRCA) College of Pharmacy Pusan National University Busan 46241 Korea; ^2^ Research Center Dongnam Institute of Radiological & Medical Sciences Busan Korea; ^3^ Pathological and Analytical Center Korea Institute of Toxicology 141 Gajeong‐ro, Yuseong‐gu Daejeon 34114 Korea; ^4^ Korean Medicine (KM)‐Application Center Korea Institute of Oriental Medicine (KIOM) Daegu 41062 Korea; ^5^ Department of Physiology The University of Texas Health Science Center at San Antonio San Antonio TX 78229‐3900 USA

**Keywords:** aging, inflammation, kidney, RNA, TLR7

## Abstract

We have recently reported that TLR‐related genes, including TLR7, are upregulated during aging. However, the role of TLR7 and its endogenous ligand in inflammation related to aging is not well defined. Here, we established that small RNAs trigger age‐related renal inflammation *via* TLR7 signaling pathway. We first investigated the expression changes of nine different TLRs in kidney of 6‐month‐old young rats and 20‐month‐old aged rats. The results revealed that the expression of TLR7 was the highest among nine TLRs in kidney of old rats compared to the young aged rats. Next, to assess the role of cellular RNA as a TLR7 ligand, we treated a renal tubular epithelial cell line with total RNA isolated from the kidney of young and old rats. The results showed that RNA isolated from old rats showed higher expression of TLR7, IL1β, and TNFα compared to that from young rats. Furthermore, RNA isolated from old rats induced IKKα/β/JNK/NF‐κB activation. To identify RNA that activates TLR7, we isolated small and large RNAs from old rat kidney and found that small RNAs increased TLR7 expression in cells. Finally, to investigate the local inflammatory response by small RNA, C57B/L6 mice were intraperitoneally injected with small RNAs isolated from young and old rats; thereby, RNA isolated from old rats induced higher inflammatory responses. Our study demonstrates that renal small RNAs from aged rats induce pro‐inflammatory processes *via* the activation of the TLR7/IKKα/β/JNK/NF‐κB signaling pathway, and highlights its causative role as a possible therapeutic target in age‐related chronic renal inflammation.

## Introduction

Accumulated data strongly suggest that chronic and systemic upregulation of pro‐inflammatory mediators, including C‐reactive protein (CRP), interleukin (IL)‐6, tumor necrosis factor (TNF) α, and cyclooxygenase (COX)‐2, is induced by aging and that consequently, these pro‐inflammatory molecular events contribute to the development of aging and age‐related diseases (Chung *et al*., 2009, 2011). We have recently examined age‐related differential gene expression within the rat kidney genome using next‐generation sequencing (NGS). The results showed that genes related to kidney inflammation are upregulated during aging, especially Toll‐like receptors (TLRs) and TLR signaling‐related genes. Notably, the TLR7 mRNA expression is the highest among the TLRs family (Park *et al*., 2016). Feldman *et al*. (2015) also reported the occurrence of TLR‐related inflammation during aging‐related pathology progression. Thus, these studies imply that TLR activation mediates age‐related inflammation and may contribute to the aging process as well as the development of age‐related disease.

TLRs belong to a family of pattern recognition receptors (PRRs) involved in the initiation of the innate immune system response to microbes and tissue damage (Sabroe *et al*., 2008). After recognition of their specific ligands, TLRs induce inflammatory responses by activating nuclear factor‐κB (NF‐κB) and cytokine and chemokine secretion *via* the myeloid differentiation primary response (MyD)88‐dependent or MyD88‐independent pathways (Kawasaki & Kawai, 2014). TLRs are widely expressed in the cells of many tissues, including epithelial cells, endothelial cells, dendritic cells, monocytes/macrophages, and B/T cells (Rehli, 2002). In the kidney, TLRs are expressed not only in interstitial and glomerular immune cells, but also in intrinsic renal cells such as mesangial cells, podocytes, Bowman's capsule cells, tubular epithelial cells, and renal vasculature (Roers *et al*., 2016). To date, 10 and 13 members of the human and mouse TLR families have been discovered, respectively. Among the TLR family, four members (TLR3, TLR7, TLR8, and TLR9) located in the endosome are involved in nucleic acid recognition. TLR3, TLR7, and TLR8 bind viral or bacterial RNA, whereas TLR9 binds DNA containing unmethylated CpG dinucleotides (Rehli, 2002; Sabroe *et al*., 2008; Kawasaki & Kawai, 2014; Roers *et al*., 2016).

Recent studies have shown the activation of TLR3, TLR7, and TLR8 signaling pathway responses to endogenous RNA, including mRNA, tRNA, miRNA, and long noncoding RNA (Karikó *et al*., 2004; Carpenter, 2016). Zou *et al*. (2016) reported that splenic cellular RNAs and miR‐146a are released into the circulation during polymicrobial sepsis and activate innate immune signaling *via* the TLR7 pathway. Furthermore, tumor‐released miRNA binds murine TLR7 and human TLR8 in immune cells, leading to TLR‐induced prometastatic inflammatory response that may ultimately lead to tumor metastasis (Fabbri *et al*., 2012). Moreover, RNA released from necrotic cells after ischemia–reperfusion (I/R) contributes to ischemic myocardial injury through TLR3‐Trif signaling, whereas RNase treatment leads to reduced inflammation, apoptosis, and infarction during I/R (Chen *et al*., 2014). These studies indicate that host‐derived RNA released under stress conditions mediates the inflammatory response *via* the TLR signaling pathway.

Kidney function declines with age, in association with increased levels of the cytokine IL‐6, CRP, and other markers of inflammation and oxidative stress (Bolignano *et al*., 2014). The purpose of this study is to determine the effect of RNA derived from aged rat kidney on the activation of the TLR7 signaling pathway during aging. Here, we provide evidence that small RNAs isolated from aged rat kidney increase TLR7/IKKα/β/JNK/NF‐κB pathway, generating a pro‐inflammatory state during aging. These observations clarified a previously unknown role of TLR7 in renal aging and reveal the deleterious role of small RNAs as actors within the TLR7 signaling process.

## Results

### Activation of the TLR7 signaling pathway in aged rat kidney

**Figure 1:**
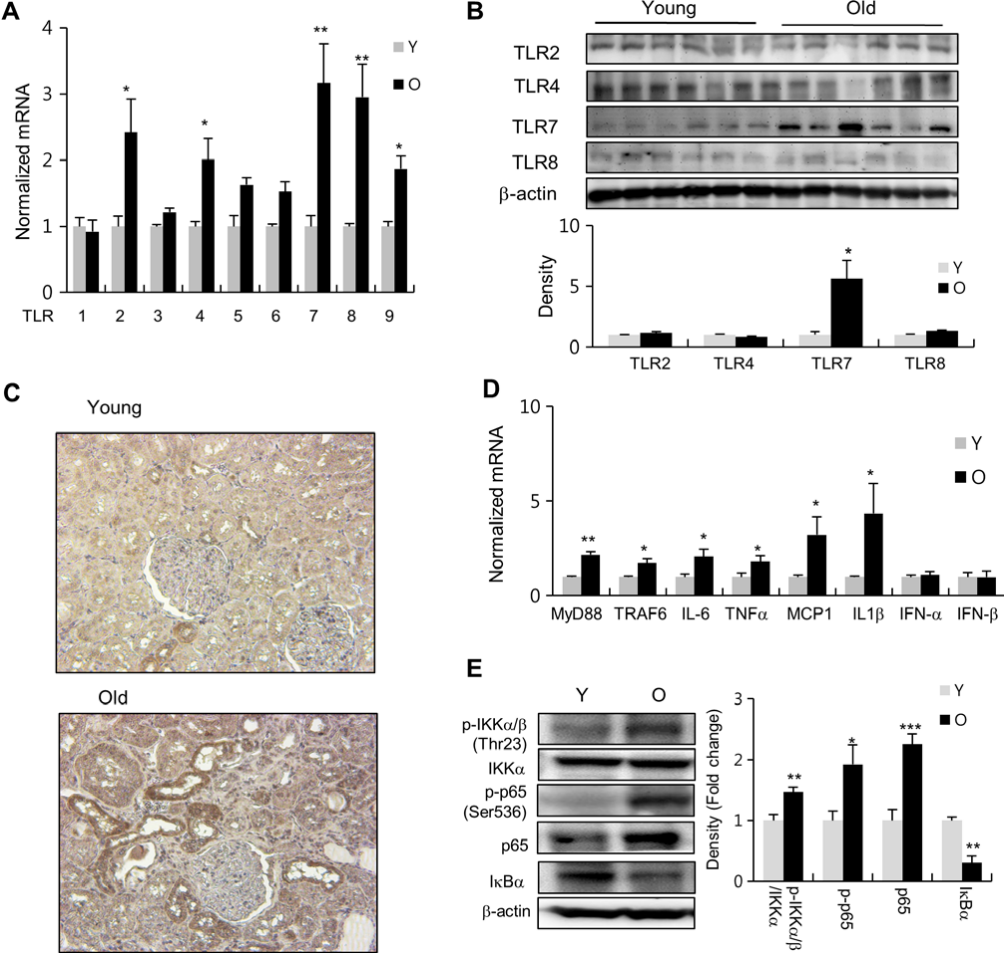
Changes in the expression of TLRs and TLR7 downstream pathway molecules in the kidney of aging rats. (A and D) RT–qPCR and (B and E) Western blotting were used to analyze the expression levels of the TLR members, TLR7 downstream molecules (MyD88, TRAF6, p‐IKKα/β, NF‐κB, and IκBα), pro‐inflammatory cytokines (IL‐6, TNFα, MCP1, and IL1β), and IFN‐α/β in young (Y) and old (O) rat kidney. The protein expression levels of TLRs, p‐IKKα/β, IKKα, p‐p65 (Ser536), p65, and IкBα were quantitated using the ImageJ software and expressed as the fold change value between young or aged rats.β‐Actin was used as a loading control. Each value is the mean ± SE. from six rats from each group (*n* = 6). *P <* *0.05, **P <* *0.01, ***P <* *0.001 vs. young rats. (C) Immunohistochemistry was used to evaluate TLR7 expression level in young and old rat kidneys. DAB‐based brown‐colored region indicates TLR7.

Our recent work using NGS analysis on aged kidney demonstrated that the TLR signaling pathway and more particularly TLR7 mRNA is highly expressed during aging (Park *et al*., 2016). To confirm the role of TLR7 in age‐related renal inflammation, we investigated changes in the expression of TLR7 and its downstream signaling molecules in aged rat kidney. We first checked both the mRNA and protein levels of TLR7, as well as other TLR family members, in both young and aged rat kidney. As shown in Fig. 1A, the mRNA expression levels of TLR2, TLR4, TLR7, TLR8, and TLR9 were significantly higher in the renal tissues of the 20‐month‐old group compared to the 6‐month‐old group, TLR7 showing the highest level among all the TLRs tested. Moreover, we investigated the differences in expression of TLR7 between young and old rats in seven tissues (kidney, muscle, liver, small intestine, spleen, heart, and aorta). The results showed that the difference in TLR7 expression between young and aged kidney was the highest among all tested tissues (Fig.  S1, Supporting information).

We then determined the changes in protein levels of TLR2, TLR4, TLR7, and TLR8 in aged rat kidney compared to those observed in young ones. As shown in Fig. 1B, the protein expression of TLR7 significantly increased in aged rat kidney compared with young kidney, whereas TLR2, TLR4, and TLR8 showed no variations.

Finally, we analyzed both the cellular location and expression levels of TLR7 in young and old rat kidney using immunohistochemical staining. The results showed that TLR7 was mainly expressed on the renal tubular epithelial cell membrane with significantly higher levels in the old rat group than in the young rat group (Fig. 1C).

Next, we investigated the expression and activation of TLR7 downstream signaling pathway molecules in young and old rat kidney using real‐time quantitative polymerase chain reaction (RT–qPCR) and Western blotting. The results showed that the mRNA levels of signaling adapter molecules, MyD88 and TNF receptor‐associated factor (TRAF) 6, and pro‐inflammatory cytokines, IL‐6, TNFα, monocyte chemotactic protein 1 (MCP1), and interleukin (IL) 1β were increased in the aged rat group compared with the young rat group (Fig. 1D). However, other TLR7 target genes, interferon (IFN)‐α/β, remained unaffected. Additionally, in the aged rat group, a downstream signaling kinase (IKKα/β) and a transcription factor (NF‐κB) were activated, while an inhibitor of NF‐κB (IκBα) was downregulated compared with the young rat group (Fig. 1E). Taken together, these data suggest that the TLR7 signaling pathway might play an important role in promoting pro‐inflammatory responses during renal aging.

### RNA from old rats increases the expression of TLR7 and inflammatory cytokines in NRK‐52E cells

**Figure 2:**
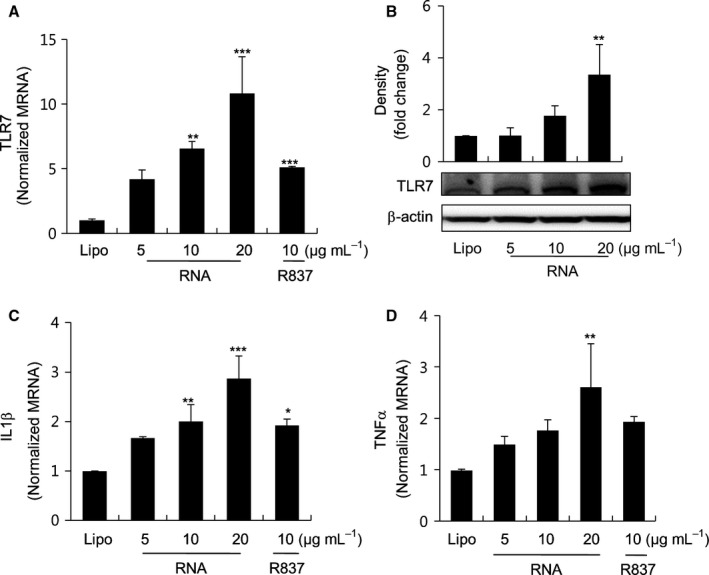
Activation of the TLR7 signaling pathway by RNA isolated from old rats. NRK52E cells were treated with RNA isolated from old rats with lipofectamine. (A, C, and D) Cells seeded in six‐well plates were treated with RNA for 3 h and collected to detect mRNA expression of TLR7, IL1β, and TNFα. (B) Cells were treated with RNA isolated from old rats for 24 h, and protein expression of TLR7 was measured by Western blotting. β‐Actin was used as a loading control. The protein expression levels of TLR7 were quantitated using the ImageJ software and expressed as the fold change over the lipofectamine‐treated control group (Lipo). Each value is the mean ± SE. *P <* *0.05, **P <* *0.01, ***P <* *0.001 vs. lipofectamine control group (*n* = 6).

To identify the molecular mechanism governing TLR7 activation in aged rat kidney, we determined whether RNA derived from old rats induces TLR7 activation in renal tubular epithelial cells NRK‐52E. First, after isolating total RNA from aged rat kidney, we treated it with lipofectamine, a liposome transfection reagent that facilitates RNA delivery in kidney cells. As shown in Fig. 2A,B, RNA of old rats increased the expression of both mRNA and protein levels of TLR7 in a dose‐dependent manner (5–20 μg mL^−1^). Furthermore, the treatment with RNA of old rats led to an increased expression of cytokine, IL1β, and TNFα in a dose‐dependent manner (5–20 μg mL^−1^) (Fig. 2C,D). The TLR7 ligand R837 was used as a positive control. Contrastingly, the mRNA expression of other TLR members (TLR2, TLR4, and TLR8) did not change after RNA treatment (Fig. S2A–C, Supporting information). Therefore, the data indicate that RNA of old rats can stimulate pro‐inflammatory responses *via* TLR7 activation in kidney.

### Effect of young and old rat RNA on the expression of TLR7 signaling pathway

**Figure 3:**
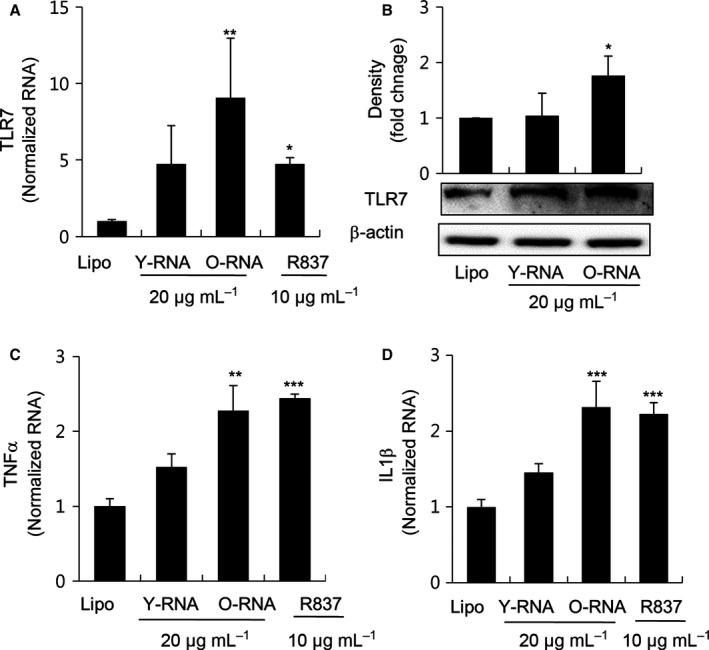
Comparison of activation levels of TLR7 signaling pathway between young and old rat RNA. NRK52E cells were treated with young and old rat RNA with lipofectamine. (A, C and D) After 3h incubation, cells were collected and the mRNA expressions of TLR7, TNFα, and IL1β were analyzed by RT–qPCR. (B) After 24h incubation, cells were collected to check protein expression of TLR7 using Western blotting. One representative result is shown, and the quantitative analysis presented beneath it. β‐Actin was used as a loading control. Each value is the mean ± SE. *P <* *0.05, **P <* *0.01, ***P <* *0.001 vs. the lipofectamine control group (Lipo, *n* = 6).

To determine the difference in TLR7 activation between young and old rat RNA, we treated total RNA isolated from young and old rats kidney on NRK52E cells, respectively. As illustrated in Fig. 3A,B, both the mRNA and protein expression levels of TLR7 were much higher after treatment with old RNA than those observed with young RNA. Furthermore, the expressions of pro‐inflammatory cytokines, TNFα and IL1β, were significantly increased in the old rats RNA treatment group (Fig. 3C,D).

### RNase and TLR7 antagonist decrease RNA‐induced TLR7 and cytokine expression

**Figure 4:**
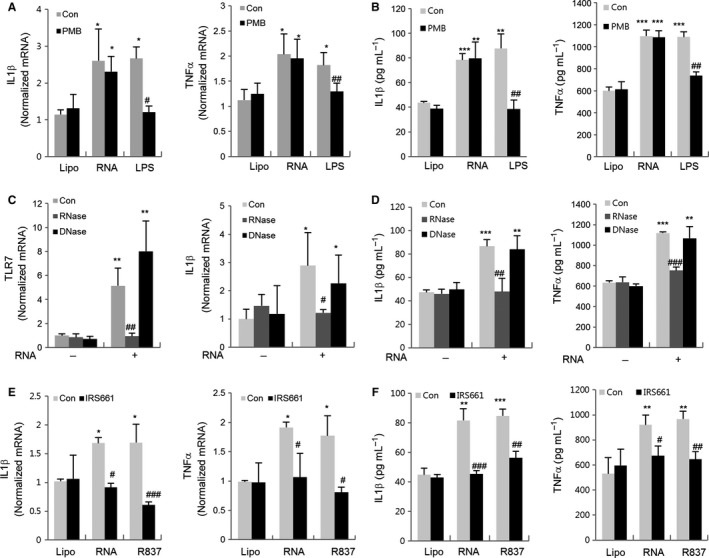
Inflammatory effects of PMB, RNase, and TLR7 inhibitor in cells treated with RNA of old rats. (A and B) RNA isolated from old rats (20 μg mL^−1^) or LPS (1 μg mL^−1^) was incubated with 50 μg mL^−1^ PMB (a LPS neutralizer) for 30 min at 4 °C before treatment in kidney cells. (C and D) RNA isolated from old rat was incubated with RNase (10 μg) or DNase (1 U) for 30 min at room temperature before treating the kidney cells. (E and F) IRS661 (2 μm) and control sequence were pretreated for 1 h in the cells before the administration of RNA isolated from old rats (20 μg mL^−1^) complexed with lipofectamine. Cells were incubated with RNA isolated from old rats (20 μg mL^−1^) for 3 h, and the expression levels of TLR7 and cytokines were analyzed by RT–qPCR (A, C, and E) and ELISA (B, D, and F). Each value is the mean ± SE. *P <* *0.05, **P <* *0.01, ***P <* *0.001 vs. the lipofectamine control group (Lipo) ^#^ *P *<* *0.05, ^##^ *P *<* *0.001, ^###^ *P *<* *0.001 vs. the LPS, RNA, and R837‐treated groups, respectively (*n* = 6).

To define the effect of RNA isolated from old rats on the TLR7 signaling pathway, we treated kidney cells with polymyxin B sulfate (PMB) (a LPS neutralizer), RNase, and TLR7 antagonist, respectively. We checked mRNA expressions for TLR7 and both mRNA and protein expressions for cytokines using RT‐qPCR and ELISA. First, to exclude the effect of any potential LPS contamination during RNA purification or treatment, the cells were incubated with total RNA isolated from old rats after PMB pretreatment. As shown in Fig. 4A,B, the PMB treatment markedly decreased the LPS‐induced upregulation of IL1β and TNFα expressions, but did not affect the expression of IL1β and TNFα induced by RNA isolated from old rats. Then, total RNA isolated from old rats was treated with RNase or DNase in the cells. The results showed that RNase treatment inhibited TLR7, IL1β, and TNFα expressions in the RNA isolated from old rats‐induced kidney cells, while DNase did not affect them (Fig. 4C,D). Finally, to investigate whether RNA isolated from old rats induces the expression of pro‐inflammatory genes through TLR7, we treated the RNA and R837 after pretreatment of IRS661, a specific TLR7 antagonist. The results showed that both RNA isolated from old rats and R837 increased the expressions of IL1β and TNFα, while IRS661 pretreatment prevented them (Fig. 4E,F).

### RNA isolated from old rats promotes TLR7 activation *via* the IKKα/β, JNK, and NF‐κB pathway

**Figure 5:**
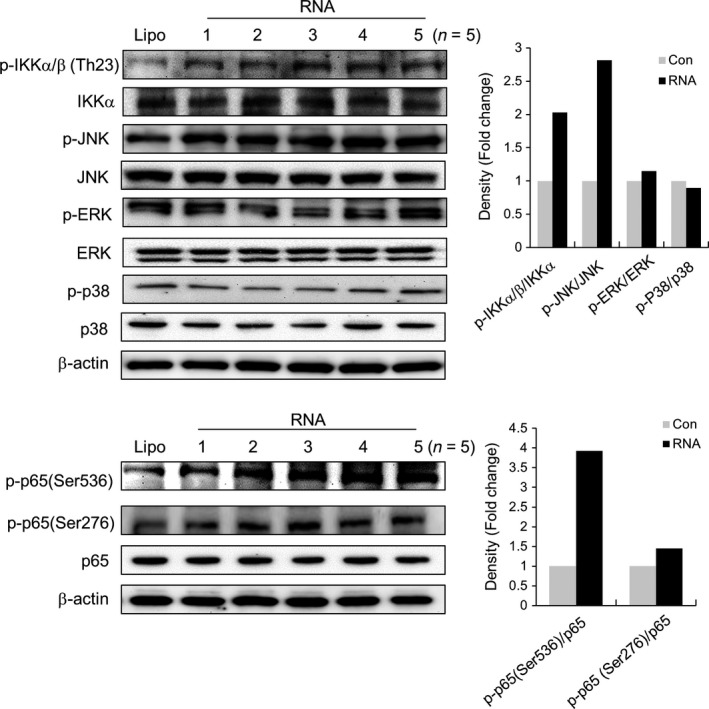
Effects of RNA isolated from old rats on IKKα/β, MAPKs, and NF‐κB activation. Kidney cells were treated with lipofectamine and lipofectamine‐complexed RNA isolated from old rats (20 μg mL^−1^) for 30 min to detect the phosphorylation of IKKα/β, JNK, ERK1/2, and p38, and total IKKα, JNK, ERK1/2, and p38 proteins using Western blotting (upper). Phosphorylation of NF‐κB (Serine 536 and 276 of p65) and total p65 protein was tested using Western blotting after 1h incubation with lipofectamine and lipofectamine‐complexed RNA (bottom). β‐Actin was used as a loading control. The protein expression levels of p‐IKKα/β, IKKα, p‐MAPKs, total MAPKs, p‐NF‐κB, and total NF‐κB were quantitated using the ImageJ software and expressed as the fold change over the lipofectamine‐treated control group (Lipo).

To determine the downstream signaling pathway of TLR7 upregulated by RNA isolated from old rats, we investigated the activation mechanisms of downstream kinases, IKKα/β, MAPKs (JNK, ERK1/2, and p38), and transcription factor NF‐κB (p65) by evaluating their phosphorylation levels. The results showed that the treatment with RNA isolated from old rats increased the phosphorylation levels of IKKα/β and JNK, but did not have any effect on ERK1/2 and p38 (Fig. 5). Moreover, RNA isolated from old rats activated NF‐κB, as evidenced by the phosphorylation of serine 536 and 276 of p65. On the other hand, the expressions of total IKKα/β, MAPKs, and p65 protein were not changed. Therefore, these data indicate that RNA isolated from old rats activates the TLR7 signaling pathway *via* IKKα/β, JNK, and NF‐κB signaling cascade.

### Aged rat kidney‐derived small RNAs promotes pro‐inflammatory activation in mice

**Figure 6:**
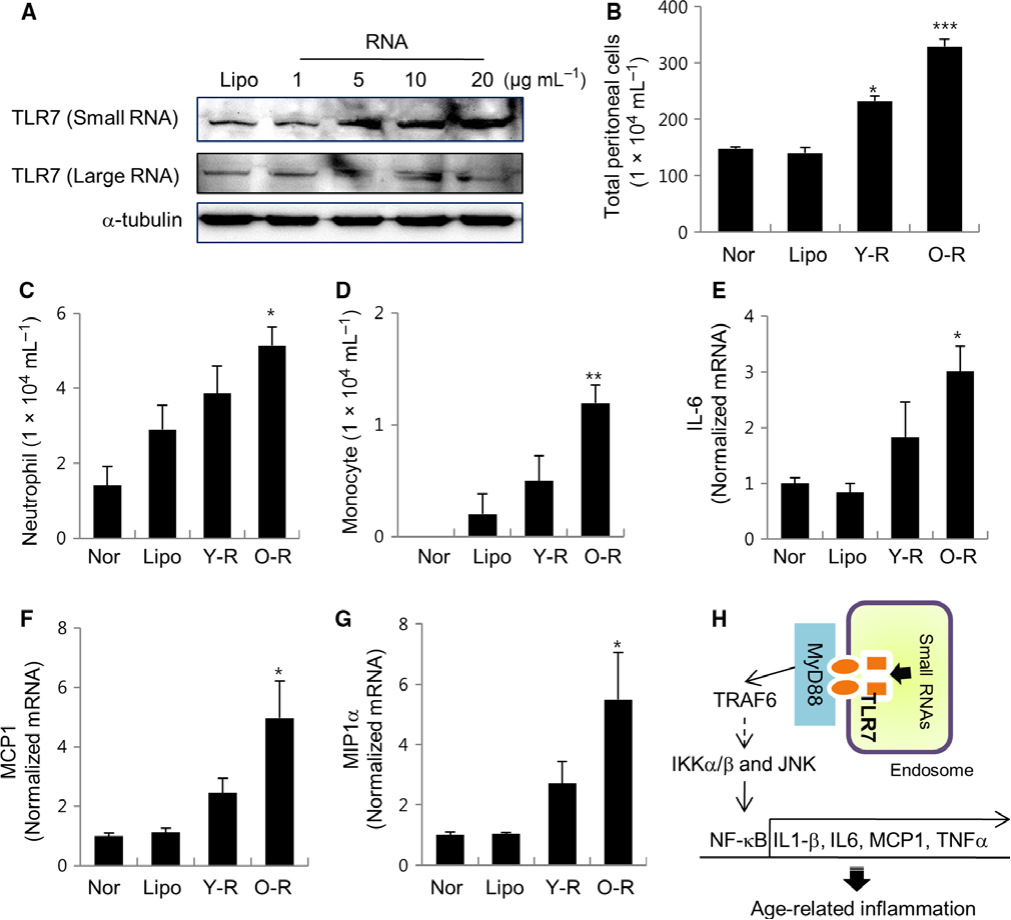
Effect of small RNAs isolated from young and old rat kidneys in the inflammatory response *in vivo*. (A) Cells were treated with small and large RNAs isolated from old rat kidney for 24 h and subsequently tested for TLR7 expression using Western blotting. α‐Tubulin was used as a loading control. (B‐G) Mice were given intraperitoneal injections with small RNAs isolated from young (Y‐R) and old (O‐R) rat kidney. Seventeen hours later, the peritoneal lavage was carried out to harvest the cells, which counted using a hemocytometer. The numbers of neutrophils and monocytes in the peritoneal cavity are determined using an ADVIA 2120i analyzer. The expressions of IL‐6, MCP1, and MIP1α were analyzed by RT–qPCR. Normal saline‐injected (Nor) and lipofectamine‐injected groups (Lipo) were used as the controls. Each value is the mean ± SE from six mice in each group. *P <* *0.05, **P <* *0.001, ***P <* *0.0001 vs. the lipofectamine control group. (H) Schematic representation of the potential mechanism by which small RNAs isolated from aged rat kidney induce TLR7 activation *via* the IKKα/β/JNK/NF‐κB pathway during aging.

To identify the type of RNA responsible for the TLR7 activation, we isolated small and large RNAs from old rat kidney and introduced them at difference concentrations (1, 5, 10, and 20 μg mL^−1^) in kidney cells. The results showed that the treatment with small RNAs from old rats at concentrations ranging from 5 to 20 μg mL^−1^ induced an increase in the expression of TLR7, whereas large RNAs did not (Fig. 6A). Therefore, this observation indicates that age‐related TLR7 expression is associated with small RNAs, but not with large one.

In preliminary study, we found that intraperitoneal injection with small RNAs from old rats into C57B/L6 mice caused local inflammatory responses through the recruitment of neutrophils and monocytes into peritoneal space (Fig.  S3, Supporting information). Next, to determine the difference in inflammatory response between young and old rat small RNAs, we injected small RNAs isolated from young and old rat kidney into mice peritoneal spaces and harvested the cells by peritoneal lavage after 17 h. As shown in Fig. 6A, the numbers of total peritoneal cells between normal mice treated by saline injection, and lipofectamine control mice was similar. Compared to the mice injected with small RNAs from young rats, the mice treated with small RNAs from old rats showed a higher increase in the number of total peritoneal cells (Fig. 6B). In addition, the small RNAs isolated from old rats displayed significant increase in the recruitment of neutrophils and monocytes into the peritoneal space in mice compared to small RNAs isolated from young rats, suggesting an inflammatory cell influx into the peritoneal space (Fig. 6C,D). More particularly, the small RNAs from old rats induced a marked increase in not only inflammatory cells migration, but also gene expressions of IL‐6, MCP1, and MIP1α in migrated immune cells compared to the small RNA isolated from young rats (Fig. 6E–G). Therefore, the results suggest that small RNAs from old rats are capable of mediating the local activation of immune cells *in vivo*.

## Discussion

In the current study, we found that small RNAs isolated from old rat kidneys increase the pro‐inflammatory TLR7 activation *via* the IKKα/β/JNK/NF‐κB pathway during aging. Previously, we reported that TLR signaling‐related molecules are increased in kidney of 25‐month‐old rats based on RNA whole‐genome sequencing analysis (Park *et al*., 2016). As a follow‐up, in the present study, we further explored the role of TLR7 in renal aging and aimed at determining whether RNA isolated from old rats can function as a novel endogenous ligand of TLR7 and thereby induce its activation in kidney cells. The results revealed that treatment of cultured NRK52E cells with total RNA isolated from old rats induced a significant increase in the expressions of TLR7 and cytokines. Our data revealed that total RNA isolated from old rats increases considerably TLR7 activation compared to that of young rats. And we have also shown that, the increase of TLR7 activation by total RNA isolated from old rats was mediated through IKKα/β/JNK/NF‐κB activation. Moreover, among the total RNA, small RNAs isolated from aged rat kidney increased TLR7 expression, while large RNAs did not. Finally, small RNAs isolated from old rat kidney led to a significant increase in local inflammatory responses after its peritoneal injection into mice when compared to inflammatory responses observed with small RNAs isolated from young rat injected into mice.

Therefore, it appears that renal small RNAs isolated from old rats enhanced the pro‐inflammatory activation *via* the TLR7 signaling pathway during aging, which may play an important role in age‐related chronic inflammation of kidney.

The innate immune system is the first line of defense against pathogen RNA (Jensen & Thomsen, 2012). However, several recent studies have reported that host‐derived cellular RNA can function as a danger molecule, provoking an inflammatory response *via* TLR signaling. Feng *et al*. (2015) reported that cellular RNA isolated from cardiac cells or tissues, or extracellular RNA released from injured cardiomyocytes, induces pro‐inflammatory responses in cardiomyocytes and immune cells *via* TLR7‐MyD88 signaling. Also, splenic RNA and miR‐146a are released into the circulation during polymicrobial sepsis and promote the production of complement factor B *via* TLR7 signaling (Zou *et al*., 2016). In addition, UVB irradiation‐mediated production of U1 small nuclear RNA induces cytokine production *via* TLR3 in keratinocytes (Bernard *et al*., 2012). Our results are consistent with the conclusion of these studies, suggesting that old rat kidney‐derived RNA plays a pivotal role in mediating TLR7‐induced inflammation during aging.

In our study, we found that small RNAs, among the total RNA isolated from old rat kidney, contribute to TLR7‐mediated pro‐inflammatory responses. Small RNAs show sizes below 200 nucleotides and are usually noncoding RNA molecules such as miRNA, piwi‐interacting RNA, small nucleolar RNA, tRNA‐derived small RNAs, and small nuclear RNA that are key regulators of gene expression (An & Song, 2011). Small RNAs are known to modulate numerous pathways (insulin pathway, DNA damage response, mitochondrial function, and cell death) related to aging and aging‐related inflammatory diseases (Smith‐Vikos & Slack, 2012; Dhahbi, 2014; Rippo *et al*., 2014). Our study shows that the administration of small RNAs from old rat kidneys into the peritoneal cavity of mice increases the occurrence of acute peritonitis significantly, as evidenced by a marked migration of immune cells into the peritoneal space and an increased expression of cytokines by migrated immune cells. Therefore, small RNAs from old rats may be used as powerful anti‐aging therapeutic targets toward the fine‐tuning of target proteins, and our next study will focus on determining the specific types of small RNAs involved and their roles during inflammation, at a molecular level.

The molecular mechanisms by which the mammalian immune system senses self‐nucleic acids are not fully understood. However, a few mechanisms have been proposed. mouseTLR7 and humanTLR8 tend to recognize specific uridine‐ or uridine/quanosine‐rich sequences on RNA (Heil *et al*., 2004). Fabbri *et al*. (2012) showed that the GU motif in the 18–21 nucleotide sequences of miR‐21 and miR‐29a is involved in TLR activation. Also, endosome TLRs, namely TLR3, TLR7, and TLR8, prevent the recognition of self‐RNA under normal physiological conditions, whereas self‐RNA in extracellular vesicles such as exosome, microvesicle, or phagosome, which is released under stress conditions, stimulates the immune response (Brencicova & Diebold, 2013). It has been reported that RNA that has not been treated with any liposome transfection agents, such as lipofectamine, fails to induce cytokine production in cardiomyocytes (Feng *et al*., 2015). Thus, liposome‐mediated uptake of cellular RNA may mimic *in vivo* situations, such as the fusion of extracellular vesicles, to enable the interaction between cells and exRNA released from stressed cells.

Several studies have revealed that TLRs are associated with aging and age‐related inflammatory diseases such as chronic kidney disease (CKD), cardiovascular diseases, and Alzheimer's disease (Yiu *et al*., 2014). Aging‐associated kidney disease is usually considered as a degenerative process associated with aging and affected by various inflammatory factors, including the renin angiotensin system, endothelial nitric oxide, and oxidative stress (Okamura & Pennathur, 2015). TLRs have been shown to play an important role in the innate immune system by triggering pro‐inflammatory signaling pathways in response to renal injury (Robson, 2009). Increased TLR4 expression contributes to renal fibrosis and CKD progression *via* inflammasome activation in renal epithelial cells (Souza *et al*., 2015). Additionally, in nephritic lesions of MRL^lpr/lpr^ mice, TLR7 is expressed on renal macrophages and contributes to the production of pro‐inflammatory mediators, including IL‐12, IL‐6, CCL2, and IFN‐α, which is known to contribute to the progression of lupus nephritis (Pawar *et al*., 2006). Interestingly, TLR7 transgenic mice die prematurely and show immune deposits in the kidney and severe hepatic injury, but overexpression of RNase in TLR7 transgenic mice results in an increased survival and protection from inflammation in kidney and liver (Sun *et al*., 2013). Our results also showed the difference in expression of TLR7 between young and old rats is higher in kidney than in other tissues including muscle, spleen, small intestine, liver, heart, and aorta. Therefore, the current results indicate that TLR7 is a crucial factor in the aging process of kidney and may provide a mechanistic support for toward understanding of close link between aging and inflammation.

In summary, this study indicates that small RNAs derived from aged rat kidney bind TLR7, and the activated TLR7 induces an increase in pro‐inflammatory responses *via* the IKKα/β/JNK/NF‐κB signaling pathway during aging (Fig. 6H).

In conclusion, this study presents the evidence for the role of small RNAs from old rats in the enhanced activation of TLR7 during renal aging. The main significance of our study resides in the fact that cellular RNAs isolated from aged rat kidney can act as signals for TLR7 activation during aging. Notably, our study showed for the first time that small RNAs from old rats are potential endogenous ligands involved in the TLR7‐induced inflammatory response during renal aging and further mediates acute innate immune response through their role as inflammatory mediators when injected into a mouse's peritoneal cavity. Collectively, these data strongly indicate that small RNAs from old rats can act as novel endogenous danger molecules during renal aging and may play an important role in mediating age‐related chronic inflammation.

## Materials and methods

### Material

All chemical reagents, except when specified, were obtained from Sigma‐Aldrich (St. Louis, MO, USA), including PMB, LPS (*Escherichia coli* O26:B6), and RNase A. DNase was purchased from New England Biolabs Inc. (Beverly, MA, USA). Imiquimod (R837, TLR7 ligand) was obtained from Invivogen (San Diego, CA, USA). Specific immunoregulatory DNA sequences (IRS) were synthesized as TLR7 antagonists by Bioneer Inc. (Daejeon, Korea). The following sequences were used: IRS661 (TLR7 inhibitor: 5′‐TGCTTGCAAGCTTGCAAGCA‐3′), and control oligonucleotide (5′‐TCCTGGCGGAAAAGT‐3′). Antibodies against TLR2, TLR4, p‐IKKα/β, IKKα, p‐p65 (Ser536), p‐p65 (Ser276), p65, IκBα, β‐actin, p‐JNK, p‐ERK, and p‐p38 were obtained from Santa Cruz Biotechnology (Dallas, TX, USA). The TLR7 antibody was obtained from Novus Biologicals Inc. (Littleton, CO, USA) and antibodies against ERK, JNK, and p38 were purchased from Cell Signaling Technology, Inc. (Danvers, MA, USA). All other materials were obtained in the highest available grade.

### Young and old rats for kidney samples

Male Sprague Dawley rats, 6 and 20 months old, were obtained from Samtako (Osan, Korea). Animals were housed individually in polycarbonate cages with wood chip bedding and were maintained in an air‐conditioned animal room (temperature: 24°C, relative humidity: 55 ± 5%) on a 12h light/dark cycle. Rats at 6 and 20 months of age were anesthetized and their kidney was quickly removed and rinsed in ice‐cold saline. The renal tissue of the upper pole of the kidney was placed into 10% neutral formalin for fixation and used for immunohistochemical analysis. The remaining kidney tissue was stored −80°C for subsequent Western blotting and RT–qPCR analysis. The animal study was designed by the Aging Tissue Bank and approved by the Pusan National University—Institutional Animal Care and Use Committee (Approval Number PNU‐2014‐0601).

### Immunohistochemical analysis

Kidney was fixed in 10% neutral formalin and processed for paraffin‐embedding according to standard procedures. Sections were 4 μm thick. Immunohistochemical analysis was performed using a TLR7 (Novus Biologicals Inc.) antibody. Formalin‐fixed, paraffin‐embedded kidneys were stained using a commercial kit (DAB‐based IHC systems, Invitrogen, Carlsbad, CA, USA) according to the manufacturer's instructions.

### Kidney tissue homogenates and Western blotting

Tissue pieces (0.1 g) were added into a 2‐mL centrifuge tube containing stainless beads. CETi lysis buffer (Translab, Daejeon, Korea) was immediately added and subjected to oscillation using the TissueLyser II machine (Qiagen, Hilden, Germany) at 30 Hz for 3 min. The lysate was incubated for 15 min on ice and then centrifuged at 12 000 *g* for 10 min, at 4°C. The supernatant was retained and used for BCA assay (Thermo Fisher Scientific Inc., Waltham, MA). The samples were prepared in gel loading buffer [0.125 m Tris–HCl, pH 6.8, 4% sodium dodecyl sulfate (SDS), 10% 2‐mercaptoethanol, and 0.2% bromophenol blue] at a volume ratio of 1:1 and were boiled for 5 min. Western blotting was performed as described previously (Lee *et al*., 2013).

### RNA extraction and RT–qPCR

Total RNA was extracted from rat kidney tissue (0.1 g) using RiboEx (GeneAll, Seoul, Korea) and was resolved in sterilized RNase‐free water. The RNA concentration was determined using a NanoDrop spectrophotometer (Thermo Fisher Scientific Inc.), and the purified RNA was subsequently aliquoted and stored at −80°C. Small and large RNAs were extracted using a Hybrid‐R miRNA purification kit (GeneAll). To perform RT–qPCR, cDNA was synthesized using amfiRivert platinum cDNA synthesis master mix (GenDEPOT, Barker, TX, USA). The synthesized cDNA was used as a template for RT–qPCR, which was performed using SYBR green real‐time master mix (Bioline, London, UK). RT–qPCR and data analyses were performed using the CFX Connect System (Bio‐Rad Laboratories Inc., Hercules, CA, USA). Primer sequences are shown in Supplemental Table 1.

### Cell culture and RNA treatment

NRK52E cells, from a renal tubular epithelial cell line, were obtained from American Type Culture Collection (ATCC; American Type Culture Collection, Manassas, VA, USA). The cells were grown in Dulbecco's modified eagle medium (GenDEPOT) containing 2 mm l‐glutamine, 100 units mL^−1^ penicillin, 100 μg mL^−1^ streptomycin, and 10% heat‐inactivated fetal bovine serum (GenDEPOT). Cells were maintained at 37°C in a humidified atmosphere containing 5% CO_2_. To perform the transfection of RNA, the media of cultured cells were replaced with prewarmed fresh culture media before treatment. Purified RNA was complexed with lipofectamine 3000 (Life Technologies, Grand Island, NY, USA) following the manufacturer's instructions. For additional nuclease predigestion experiments, RNA was incubated with RNase (10 μg) or DNase (1 U) at room temperature for 30 min before being complexed with lipofectamine 3000. For the TLR7 inhibition experiments using oligonucleotides, the cells were treated for 1 h with 2 μm of IRS661 or control sequence, previously complexed with lipofectamine 3000, before RNA treatment.

### TNFα and IL1β measurement

The RNA‐treated NRK52E cells were lysed for 20 min on ice in RIPA buffer containing protease inhibitor cocktail and then centrifuged at 14 000 *g* at 4°C for 10 min. Then, the supernatant was collected. The total protein concentration was determined by BCA method. The expression of TNFα and IL1β in cells was measured by ELISA kits (KOMA BIOTECH, Seoul, Korea) according to manufacturer's instructions.

### *In vivo* RNA administration experimentation

Male (10 weeks old) C57BL/6 mice were purchased from Samtako and acclimated for 1 week prior to carrying out the research study. Mice were injected intraperitoneally with saline, lipofectamine, or lipofectamine‐complexed small RNAs isolated from young and old rat kidney (60 μg per mouse), and 17 h later, cells from their peritoneal cavities were harvested with 11 mL of phosphate‐buffered saline (PBS), and centrifuged. The pellets were resuspended in PBS, and the cells subsequently were counted using a hemocytometer to determine the number of total peritoneal cells. The numbers of neutrophils and monocyte among the total peritoneal cells were determined using an ADVIA 2120i analyzer (ADVIA 2120i Siemens, New York, NY, USA). Total RNA was isolated from peritoneal cells for gene expression assay.

### Statistical analysis

Values are shown as mean ± SE. Analyses were performed using graphpad prism 5 (GraphPad software, La Jolla, CA, USA). Statistical differences between young and old group were determined by the Student *t*‐test. The statistical significance of differences between multiple tests was determined by a one‐factor analysis of variance (ANOVA) followed by a Bonferroni multiple comparison test. Values of *P *<* *0.05 were considered statistically significant.

## Funding

This work was supported by a National Research Foundation of Korea (NRF) grant funded by the Korea Government (MSIP) (No. 2009‐0083538). This research was also supported by Basic Science Research Program through the National Research Foundation of Korea (NRF) funded by the Ministry of Education (No. 2014R1A1A2008973).This research was supported by Bio & Medical Technology Development Program of the National Research Foundation of Korea (NRF) funded by the Ministry of Science, ICT & Future Plannig (No. 2015M3A9B8029074).

## Author contributions

E.K. Lee, D.H. Kim, B. Lee, K. J. Jung, and H.Y. Chung involved in conception and design of study; E.K. Lee, K.W. Chung, R. Kim, S Ha, and S.D. Kim involved in helping with an experiment; E.K. Lee, K.W. Chung, Y. R. Kim, S.D. Kim, D.H. Kim, B. Lee, K.J. Jung, and H.Y. Chung involved in analysis and/or interpretation of data; E.K. Lee, B.P. Yu, and H.Y. Chung involved in drafting the manuscript; E.K. Lee, K.W. Chung, Y. R. Kim, D.H. Kim, B. Lee, K. J. Jung, B.P. Yu, and H.Y. Chung involved in revising the manuscript critically for important intellectual content.

## Conflict of interest

The authors declare that they have no competing interests.

## Supporting information


**Fig. S1** Effect of aging on the expression of TLR7 in different types of tissues.

**Fig. S2** Effect of RNA isolated from old rat kidney on the expression of TLR2, TLR4 and TLR8.

**Fig. S3** Induction of the inflammatory response by small RNAs isolated from old rats *in vivo*.

**Table S1** Primers used in RT–qPCR.
