# Supplementary figures and images for: Small RNAs induce the activation of the pro‐inflammatory TLR7 signaling pathway in aged rat kidney
Source: Aging Cell. 2017 Jun 30;16(5):1026–34. doi: 10.1111/acel.12629 (PMC5595700; doi:10.1111/acel.12629)

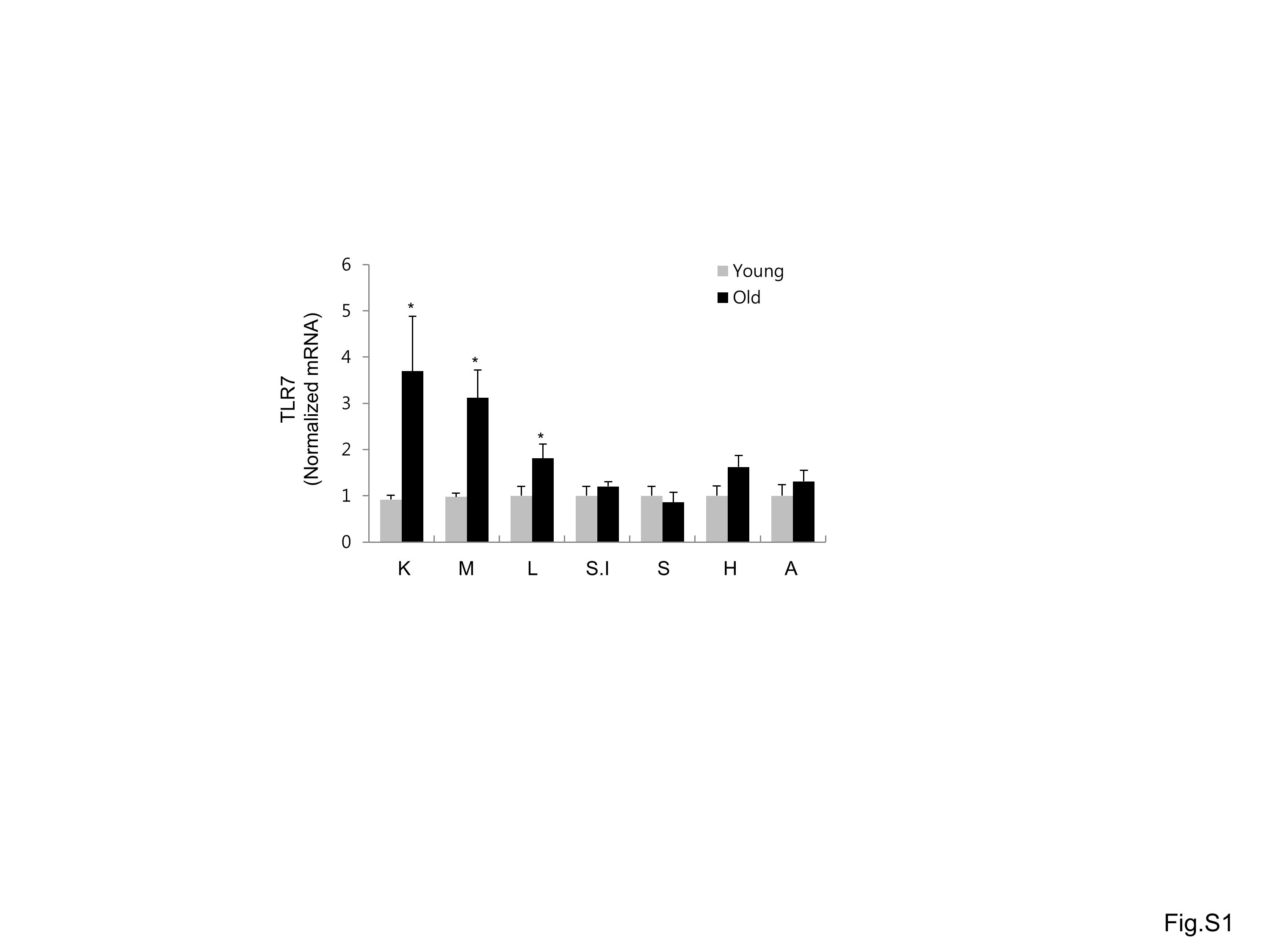

Supplement: Supplementary file 1 — Fig. S1 Effect of aging on the expression of TLR7 in different types of tissues. [file ACEL-16-1026-s001.tif]

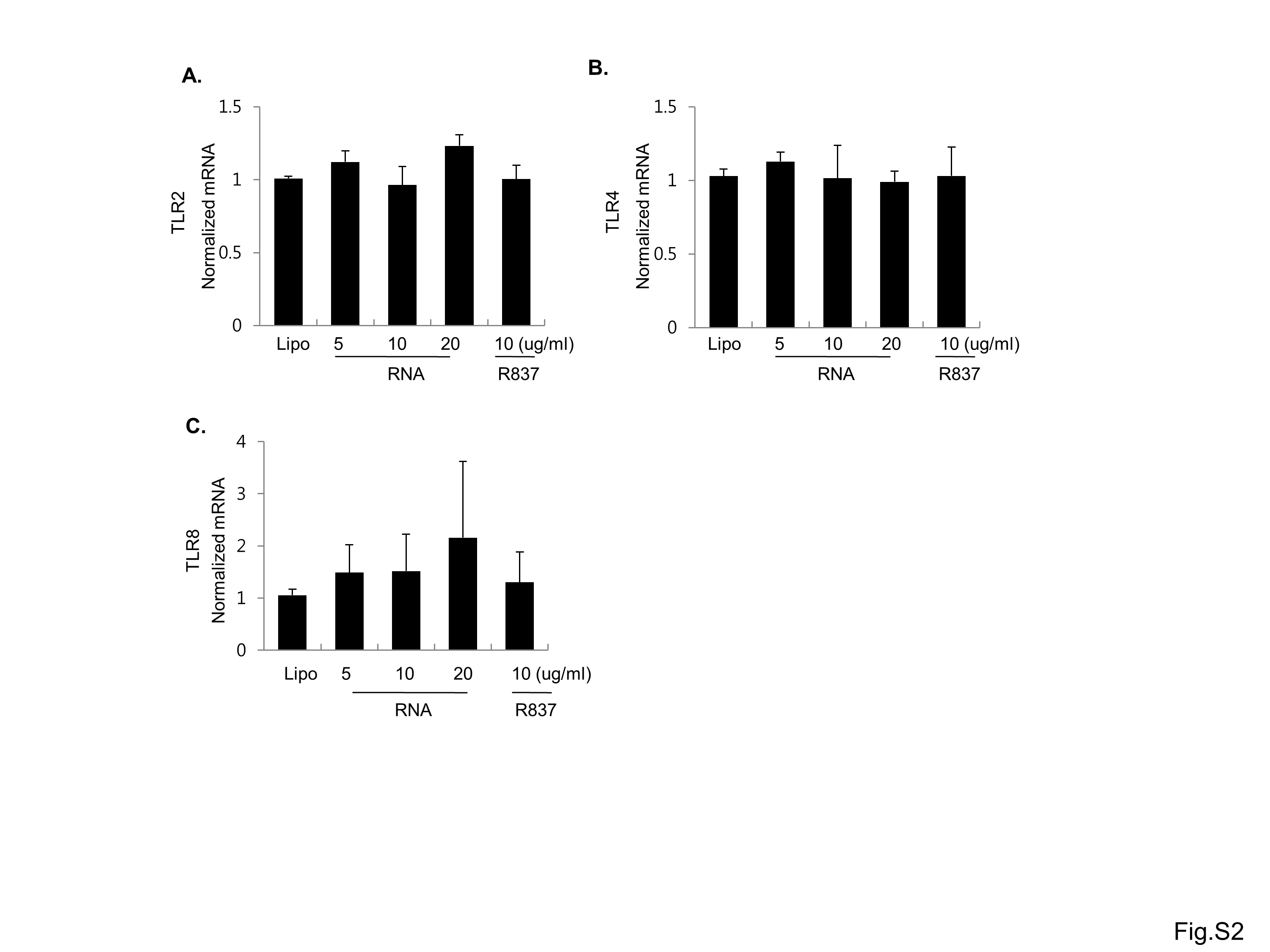

Supplement: Supplementary file 2 — Fig. S2 Effect of RNA isolated from old rat kidney on the expression of TLR2, TLR4 and TLR8. [file ACEL-16-1026-s002.tif]

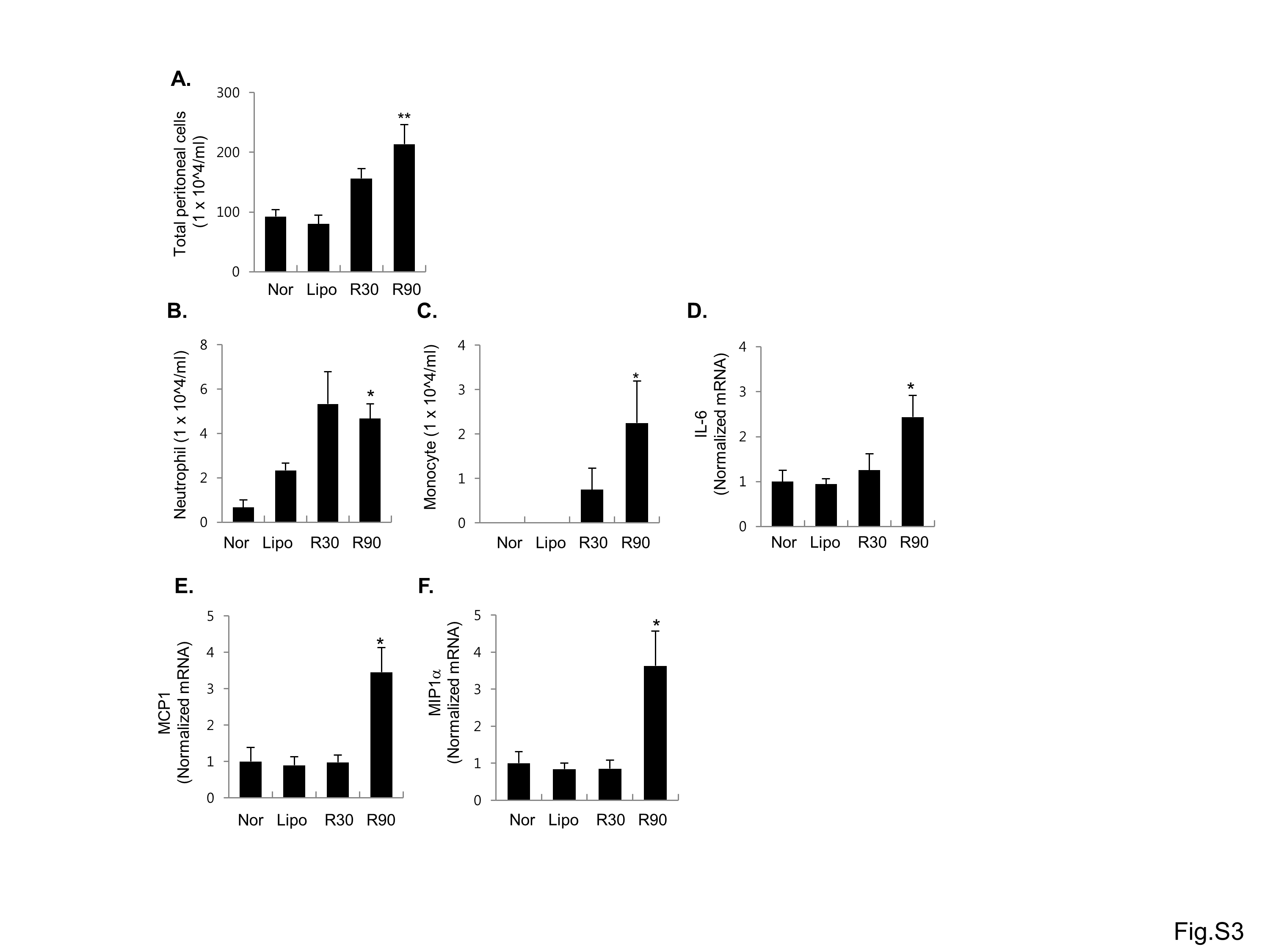

Supplement: Supplementary file 3 — Fig. S3 Induction of the inflammatory response by small RNAs isolated from old rats in vivo. [file ACEL-16-1026-s003.tif]

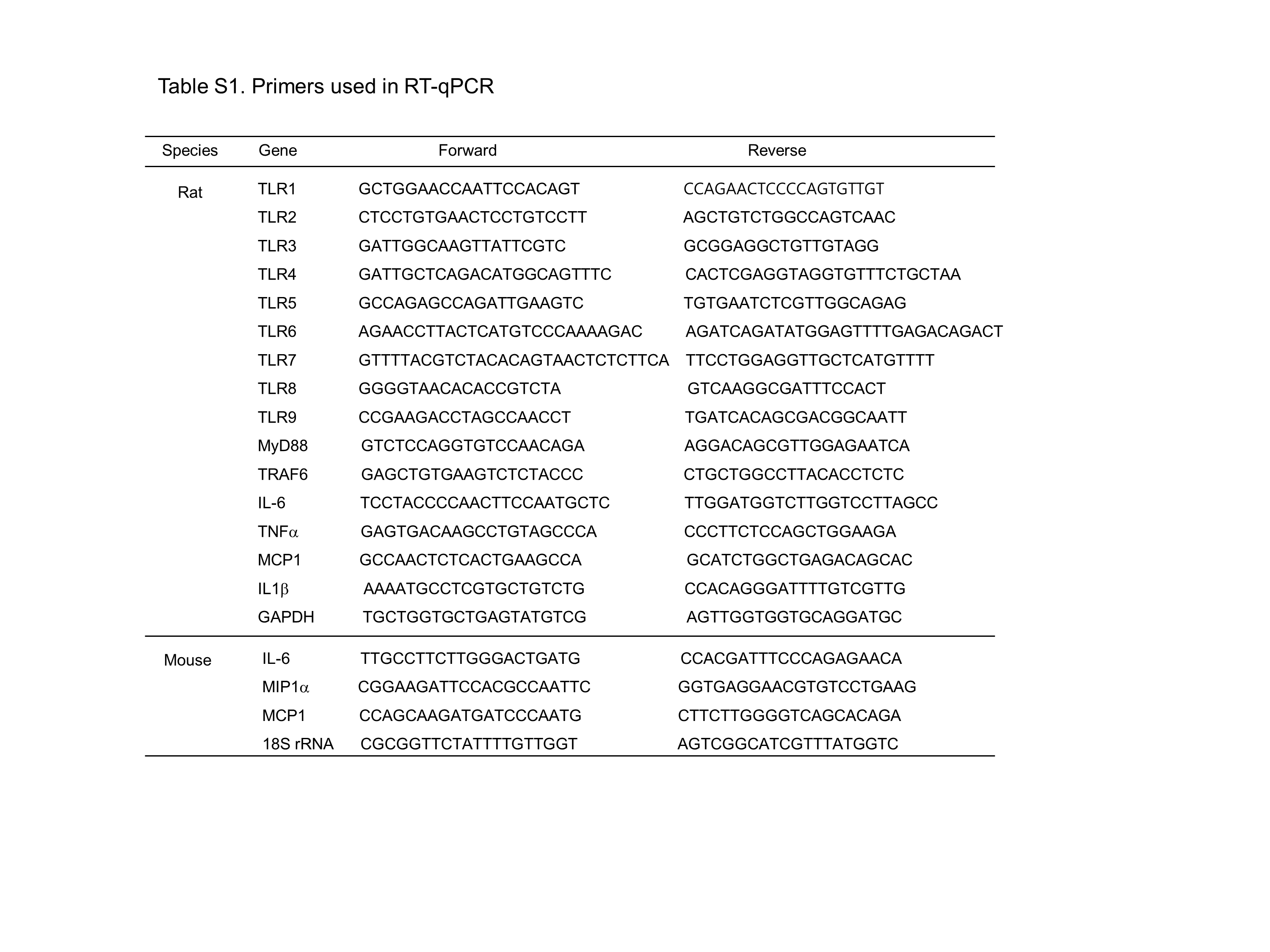

Supplement: Supplementary file 4 — Table S1 Primers used in RT–qPCR. [file ACEL-16-1026-s004.tif]
